# Associations between adiposity measures and depression and well-being scores: A cross-sectional analysis of middle- to older-aged adults

**DOI:** 10.1371/journal.pone.0299029

**Published:** 2024-03-06

**Authors:** Caoimhe Lonergan, Seán R. Millar, Zubair Kabir

**Affiliations:** School of Public Health, University College Cork, Cork, Ireland; Saga University, JAPAN

## Abstract

**Background:**

Obesity and mental health are significant global health concerns. Evidence has linked increased adiposity with depression and well-being; however, there is limited documented evidence in Ireland. Research also suggests lifestyle factors and disease conditions to be related to mental health. These may modulate relationships between adiposity and depression and well-being.

**Methods:**

This was a cross-sectional study of 1,821 men and women aged 46–73 years, randomly selected from a large primary care centre. Depression and well-being were assessed using the 20-item Centre for Epidemiologic Studies Depression Scale (CES-D) and the World Health Organization-Five (WHO-5) Well-Being Index. Linear regression analyses were performed to examine relationships between mental health scores (dependent variable) and adiposity (independent variable) defined using body mass index (BMI) and waist-height ratio while adjusting for demographic characteristics, lifestyle factors and disease conditions.

**Results:**

BMI and waist-height ratio had a significant positive association with depression scores and a significant inverse association with well-being scores in males and females. These associations were maintained following adjustment for demographic variables and lifestyle factors. In final models where disease conditions were adjusted for, BMI (β = 0.743, *p* < .001) and waist-height ratio (β = 0.719, *p* < .001) associations with the CES-D score remained significant. In stratified analyses, relationships between measures of adiposity and depression were found to be stronger in females (BMI: β = 0.806, *p* = .007; waist-height ratio: β = 0.768, *p* = .01) than males (BMI: β = 0.573, *p* = .049; waist-height ratio: β = 0.593, *p* = .044) but no effect modification was identified.

**Conclusions:**

These findings suggest that increased adiposity is significantly associated with poorer mental health, independent of lifestyle factors and disease conditions. Targeted interventions for reducing depression should include better population-level weight management measures.

## Introduction

Obesity and mental health contribute significantly to the global burden of diseases [1]. The obesity epidemic is ongoing in many parts of the world and in Ireland obesity and obesity-related illnesses have been projected to cost healthcare systems €5.4 billion by 2030 [2]. In 2019, the World Health Organization reported that more than one in every 100 deaths were due to suicide (1.3%) [3] and noted depression to be one of the most relevant risk factors [4]. Ireland has one of the highest rates of mental health illness in Europe, ranking third out of 36 countries [5], and the Healthy Ireland Framework has estimated that mental health issues cost the Irish economy €11 billion every year [6].

Evidence suggests that increased adiposity and mental illness are clinically related [7–12]. An obesity prevalence of 57.8% was reported in a cohort with severe depression [13] and majorly depressed individuals were shown to be 1.2 to 1.5 times more likely to suffer from obesity compared to non-depressed individuals [14]. However, there are conflicting findings; one longitudinal study from a 12-year population health survey had mixed conclusions and found that while obesity is a meaningful predictor of depression among adult males, it is not significant when predicting depression in adult females [15]. In addition, a systematic review of epidemiological studies concluded that the overall quality of evidence linking increased adiposity with depression was unsatisfactory due to potential bias and a lack of diversity among the populations examined [16].

Multifactorial processes are thought to contribute to psychological health [17]. It is well established that certain modifiable lifestyle factors are related to both physical and mental health. The third national Survey of Lifestyle, Attitudes and Nutrition (SLÁN) in Ireland (n = 10,364) showed that moderate alcohol intake, being physically active and higher dietary quality are associated with better mental health among free-living individuals aged 18 years and over [18]. Another study demonstrated that a combination of healthy lifestyle behaviours (never smoker, moderate alcohol consumption, moderate or high levels of physical activity and healthy diet) was associated with psychological health in middle- to older-aged adults; subjects with the fewest healthy behaviours (zero or one) were twice as likely to have depression compared to those with four [19]. Importantly, these lifestyle factors are also known to be related to overweight and obesity. In addition, increased adiposity is associated with chronic conditions including type 2 diabetes, cardiovascular disease and many cancers [14], which in turn can lead to an increase in depressive symptoms and poorer well-being [19].

As the causal pathway is unclear, it is important to examine relationships between adiposity and depression and well-being which take into account their shared risk factors. In addition, it is essential to demonstrate reproducibility and consistency in these relationships among different populations using different measures of adiposity. Therefore, the aim of this study was to examine associations between mental health scores and adiposity defined using body mass index (BMI) and waist-height ratio to determine whether significant relationships persist following adjustment for lifestyle factors and common disease conditions.

## Materials and methods

### Study population and setting

The Cork and Kerry Diabetes and Heart Disease Study (Phase II–Mitchelstown Cohort) was conducted between 1^st^ May 2010 and 30^th^ April 2011 in Mitchelstown, County Cork, Ireland. A random sample was recruited from the Living Health Clinic, a primary care centre which caters for approximately 20,000 Caucasian-European individuals from urban and rural settings. Stratified sampling was used to obtain equal number of males and females from all registered attending patients in the 46–73-year age group. A total of 3,807 subjects were selected. A number of these potential participants were excluded due to deaths, duplicates and incapacity to consent or attend appointments. Of the 3,051 subjects who were invited to take part in the study, 2,047 (49% male) completed the questionnaire and physical examination components of the baseline assessment (response rate: 67%). Details in relation to the study design, sampling procedures and methods of data collection have been previously reported [20].

Ethics committee approval conforming to the Declaration of Helsinki was obtained from the Clinical Research Ethics Committee of University College Cork. A letter signed by the contact GP in the clinic was sent out to all selected participants with a reply slip indicating acceptance or refusal. All participants provided written consent to use their data for research purposes.

Data on mental health and anthropometric measurements were available for 1,821 subjects. We accessed collected data in April 2023; all data were fully anonymised and we had no access to information that could identify individual participants.

### Clinical procedures

Study participants attended the clinic in the morning after an overnight fast and blood samples were taken on arrival. Fasting glucose and glycated haemoglobin A_1c_ (HbA_1c_) concentrations were measured in fresh samples by Cork University Hospital Biochemistry Laboratory using standardised procedures. Glucose concentrations were determined using a glucose hexokinase assay (Olympus Life and Material Science Europa Ltd., Lismeehan, Co. Clare, Ireland) and HbA_1c_ levels were measured in the haematology laboratory on an automated high-pressure liquid chromatography instrument Tosoh G7 [Tosoh HLC-723 (G7), Tosoh Europe N.V, Tessenderlo, Belgium].

Anthropometric measurements were performed by trained researchers with reference to a standard operating procedures manual. Height was measured with a portable Seca Leicester height/length stadiometer (Seca, Birmingham, UK) and weight was measured using a portable electronic Tanita WB-100MA weighing scale (Tanita Corp, IL, USA). The weighing scale was placed on a firm flat surface and was calibrated weekly. BMI was calculated as weight in kilograms divided by the square of height in meters. Waist circumference was measured between the lowest rib and iliac crest on bare skin. Participants were instructed to breathe in, and then out, and to hold their breath while measurement was made to the nearest 0.1 cm using a Seca 200 measuring tape. The mean of two independent readings was used in analysis. Height was divided into waist circumference measurements to derive the waist-height ratio. We used two adiposity indices in our analyses as BMI is a measure of general adiposity while waist-height ratio is a measure of central adiposity. Although BMI is more frequently used in a clinical setting to assess body composition, it has been criticised as being an inaccurate measure of body fat. Both BMI and waist-height ratio do not require sex-specific cut-offs and are thus suitable for analyses which do not stratify by sex [21], as well as for cross-validation.

### Data collection

A general health and lifestyle questionnaire assessed demographic variables, lifestyle behaviours and morbidity. Information on sex, age, education, smoking status, alcohol use and diagnosis of health conditions (type 2 diabetes, cardiovascular disease and cancer) was provided by participants. Depressive symptoms and well-being were assessed using the 20-item Centre for Epidemiologic Studies Depression Scale (CES-D) [22], designed to evaluate the frequency and severity of depressive symptoms, and the World Health Organization-Five (WHO-5) Well-Being Index [23]. Higher CES-D scores indicate more severe depressive symptoms, while higher WHO-5 scores indicate greater well-being. Physical activity was measured using the validated International Physical Activity Questionnaire (IPAQ) [24].

A validated Food Frequency Questionnaire (FFQ) comprising 150 different foods was utilised for dietary assessment. The average medium serving of each food item ingested by participants over the previous year was converted into quantities using standard portion sizes. Food item quantity was expressed as (gm/d) and beverages as (ml/d). Based on the FFQ, the Dietary Approaches to Stop Hypertension (DASH) diet score was constructed. Details of the DASH score have been reported elsewhere [25]. However, to summarise, DASH is a dietary pattern rich in fruits, vegetables, whole grains and low-fat dairy foods and is restricted in sugar-sweetened foods and beverages, red meat and added fats. This diet has been promoted by the National Heart, Lung and Blood Institute (part of the National Institutes of Health, a United States government organisation) to prevent and control hypertension. In this study, DASH diet scores ranged from 11–41. Lower scores represent poorer diet quality and higher scores represent better quality diet [26].

### Classification and scoring of variables

Potential confounders considered included sex, age, education level, smoking status, alcohol intake, dietary quality, physical activity and morbidity (type 2 diabetes, cardiovascular disease and cancer). Education was defined as ‘primary level only’ and ‘secondary or higher’. Smoking status was defined as ‘current smoker’ and ‘non-smoker’. Alcohol intake was categorised as (i) non-drinker, i.e., <1 drink per week; (ii) moderate drinker, i.e. between 1 and 14 drinks per week; and (iii) heavy drinker, i.e. >14 drinks per week. Moderate drinker consumption was defined according to previous work conducted by the European Prospective Investigation in Cancer and Nutrition (EPIC) in the United Kingdom [27]. For the current analysis, these were then re-categorised as ‘moderate/non-drinker’ and ‘heavy drinker’. Physical activity was defined as low, moderate and high levels of activity using the IPAQ. Subsequently it was converted into a dichotomous variable: ‘moderate/high’ or ‘low’ physical activity. Type 2 diabetes was determined as a fasting glucose level ≥7.0 mmol/l or HbA_1c_ level ≥6.5% (≥48 mmol/mol) [28] or by self-reported diagnosis. The presence of cardiovascular disease was obtained by asking study participants if they had been medically diagnosed with any one of the following seven conditions: Heart Attack (including coronary thrombosis or myocardial infarction), Heart Failure, Angina, Aortic Aneurysm, Hardening of the Arteries, Stroke or any other Heart Trouble. Subjects who indicated a diagnosis of any one of these conditions were classified as having cardiovascular disease.

### Statistical analysis

Descriptive characteristics were examined according to depression and well-being score quartiles. Categorical features are presented as percentages and continuous variables are shown as a mean (plus or minus one standard deviation) or a median and interquartile range for skewed data. Differences across quartiles were analysed using a Pearson’s chi-square test, an ANOVA or a Kruskal-Wallis test, where appropriate. To place adiposity indices on the same scale, BMI and waist-height ratio were standardised and linear regression analyses were performed to determine adiposity measure (primary exposure of interest) associations with depression and well-being scores (outcome measures). To examine the potential confounding influence of lifestyle factors and disease conditions on the effect of adiposity on mental health, four models were run: a univariate model; a second model which adjusted for sex and age; a third model which additionally adjusted for education and lifestyle factors (smoking status, alcohol use, physical activity and dietary quality) and a fourth model which also adjusted for disease conditions (type 2 diabetes, cardiovascular disease and cancer). Effect modification was investigated using adiposity measure*sex interaction terms in the models. Further sex-stratified analyses were also undertaken. Data analysis was conducted using IBM SPSS Statistics Version 25 (IBM Corp., Armonk, NY, USA). For all analyses, a *p* value (two-tailed) of less than .05 was considered to indicate statistical significance.

## Results

### Descriptive characteristics

Table 1 displays characteristics of the study sample according to CES-D and WHO-5 score quartiles. Individuals with more severe depressive symptoms (quartile 4 compared to quartile 1 for the CES-D) and poorer well-being (quartile 1 compared to quartile 4 for the WHO-5) were significantly more likely to be female, were younger, were more likely to have type 2 diabetes and were less likely to be physically active. Participants with higher well-being scores were more likely to have a lower educational attainment and were less likely to be heavy drinkers. Significant mean differences in BMI and waist-height ratio were also observed across both CES-D and WHO-5 score quartiles.

**Table 1:** Descriptive characteristics of the study population according to depression and well-being score quartiles (n = 1821).

**Variable**	**CES-D score**				
	**Q1**	**Q2**	**Q3**	**Q4**	*p*
Male (%)	297 (53.9)	202 (47.0)	226 (51.2)	174 (43.6)	.01
Age (median)	59 (55, 64)	58 (54, 63)	59 (54, 64)	58 (54, 62)	.038
Primary education only (%)	134 (25.6)	94 (23.3)	117 (27.9)	106 (28.1)	.37
Current smoker (%)	69 (12.6)	55 (12.9)	73 (16.6)	65 (16.3)	.157
Heavy drinker (%)	294 (53.4)	240 (55.8)	224 (50.8)	210 (52.6)	.52
Low physical activity (%)	222 (42.3)	170 (40.6)	199 (47.5)	224 (58.0)	< .001
Dietary quality (mean)	27.1 ± 5.5	26.4 ± 5.4	26.5 ± 5.5	26.6 ± 5.1	.136
Type 2 diabetes (%)	37 (6.7)	30 (7.0)	32 (7.3)	59 (14.8)	< .001
Cardiovascular disease (%)	59 (10.7)	35 (8.1)	52 (11.8)	42 (10.5)	.341
Cancer (%)	21 (3.8)	13 (3.0)	24 (5.4)	13 (3.3)	.248
Body mass index, kg/m^2^ (mean)	28.1 ± 4.5	28.3 ± 4.6	28.8 ± 4.5	29.2 ± 5.1	< .001
Waist-height ratio (mean)	0.576 ± 0.07	0.576 ± 0.07	0.585 ± 0.07	0.592 ± 0.08	< .001
	**WHO-5 score**				
	**Q1**	**Q2**	**Q3**	**Q4**	*p*
Male (%)	227 (44.8)	228 (50.7)	236 (48.8)	208 (54.7)	.029
Age (median)	57 (53, 62)	58 (54, 62)	58 (54, 63)	62 (57, 66)	< .001
Primary education only (%)	127 (26.5)	93 (21.6)	116 (25.4)	115 (32.2)	.009
Current smoker (%)	77 (15.2)	74 (16.5)	65 (13.5)	46 (12.2)	.308
Heavy drinker (%)	281 (55.4)	260 (57.8)	257 (53.1)	170 (44.7)	.001
Low-level physical activity (%)	269 (55.3)	205 (46.9)	213 (45.4)	128 (35.9)	< .001
Dietary quality (mean)	26.2 ± 5.3	26.7 ± 5.3	27.0 ± 5.5	26.9 ± 5.5	.162
Type 2 diabetes (%)	59 (11.6)	33 (7.3)	36 (7.4)	30 (7.9)	.049
Cardiovascular disease (%)	50 (9.9)	51 (11.3)	46 (9.5)	41 (10.8)	.789
Cancer (%)	21 (4.1)	15 (3.3)	20 (4.1)	15 (3.9)	.911
Body mass index kg/m^2^ (mean)	29.0 ± 5.2	28.4 ± 4.8	28.2 ± 4.4	28.3 ± 4.2	.037
Waist-height ratio (mean)	0.590 ± 0.08	0.579 ± 0.08	0.575 ± 0.07	0.583 ± 0.07	.015

*p* for difference across quartiles determined from a Pearson’s chi-square test for categorical variables, an ANOVA (dietary quality, BMI and waist-height ratio) or a Kruskal-Wallis test (age).

### Linear regression

Linear regression analyses describing associations between CES-D/WHO-5 scores and BMI and waist-height ratio are shown in Tables 2 and 3. Both adiposity measures were found to be significantly associated with depression/well-being scores in univariate analyses, with BMI and waist-height ratio being positively associated with the CES-D score and inversely associated with the WHO-5 score. These significant associations were maintained following adjustment for sex, age, education and lifestyle factors. Well-being score associations with adiposity measures were attenuated in models which included disease conditions, while BMI and waist-height ratio relationships with the CES-D score remained statistically significant in fully adjusted models.

**Table 2:** Linear regression analysis of associations between body mass index and depression/well-being scores.

Depression/well-being scores	Model 1		Model 2		Model 3		Model 4	
	β (S.E.)	*P*	β (S.E.)	*P*	β (S.E.)	*P*	β (S.E.)	*P*
CES-D score	0.886 (.18)	**< .001**	1.004 (.18)	**< .001**	0.897 (.20)	**< .001**	0.743 (.21)	**< .001**
WHO-5 score	-0.365 (.12)	**.003**	-0.469 (.12)	**< .001**	-0.343 (.14)	**.011**	-0.256 (.14)	.063

Model 1: univariate.

Model 2: adjusted for sex and age.

Model 3: adjusted for sex, age, education, smoking status, alcohol use, physical activity and dietary quality.

Model 4: additionally adjusted for disease conditions (type 2 diabetes, cardiovascular disease and cancer).

Unstandardised β coefficients and standard errors (S.E) are shown. Significant *p* in **bold**.

**Table 3:** Linear regression analysis of associations between waist-height ratio and depression/well-being scores.

Depression/well-being scores	Model 1		Model 2		Model 3		Model 4	
	β (S.E.)	*P*	β (S.E.)	*P*	β (S.E.)	*P*	β (S.E.)	*P*
CES-D score	0.840 (.18)	**< .001**	1.053 (.19)	**< .001**	0.889 (.20)	**< .001**	0.719 (.21)	**< .001**
WHO-5 score	-0.266 (.12)	**.032**	-0.476 (.12)	**< .001**	-0.351 (.14)	**.009**	-0.254 (.14)	.067

Model 1: univariate.

Model 2: adjusted for sex and age.

Model 3: adjusted for sex, age, education, smoking status, alcohol use, physical activity and dietary quality.

Model 4: additionally adjusted for disease conditions (type 2 diabetes, cardiovascular disease and cancer).

Unstandardised β coefficients and standard errors (S.E) are shown. Significant *p* in **bold**.

### Sex-stratified analyses

Table 4 shows associations between BMI and depression/well-being scores stratified by sex. For both males and females, BMI was found to have a statistically significant relationship with the CES-D score once lifestyle factors and disease conditions were adjusted for. In addition, the relationship between BMI and depression was observed to be stronger in females (β = 0.806, *p* = .007) than males (β = 0.573, *p* = .049). Relationships between BMI and WHO-5 well-being scores were attenuated and non-significant after adjustment for lifestyle factors, in both sexes.

**Table 4:** Linear regression analysis of associations between body mass index and depression/well-being scores–stratified by sex.

Depression/well-being scores	Model 1		Model 2		Model 3		Model 4	
**Males**	β (S.E.)	*P*	β (S.E.)	*P*	β (S.E.)	*P*	β (S.E.)	*P*
CES-D score	0.880 (.25)	**< .001**	0.895 (.25)	**< .001**	0.688 (.28)	**.014**	0.573 (.29)	**.049**
WHO-5 score	-0.446 (.17)	**.01**	-0.475 (.17)	**.006**	-0.297 (.19)	.119	-0.198 (.20)	.316
**Females**	β (S.E.)	*P*	β (S.E.)	*P*	β (S.E.)	*P*	β (S.E.)	*P*
CES-D score	1.028 (.27)	**< .001**	1.095 (.27)	**< .001**	1.029 (.29)	**< .001**	0.806 (.30)	**.007**
WHO-5 score	-0.383 (.18)	**.029**	-0.468 (.17)	**.007**	-0.353 (.19)	.062	-0.260 (.19)	.177

Model 1: univariate.

Model 2: adjusted for age.

Model 3: adjusted for age, education, smoking status, alcohol use, physical activity and dietary quality.

Model 4: additionally adjusted for disease conditions (type 2 diabetes, cardiovascular disease and cancer).

Unstandardised β coefficients and standard errors (S.E) are shown. Significant *p* in **bold**.

Linear regression models demonstrating associations between waist-height ratio and depression/well-being scores according to sex are shown in Table 5. Similar findings were observed, with the CES-D score showing a statistically significant association with waist-height ratio in both sexes and that the positive relationship between adiposity and depression was stronger in females (β = 0.768, *p* = .01) than males (β = 0.593, *p* = .044). For both male and female study participants, associations between waist-height ratio and the WHO-5 well-being index were non-significant in fully adjusted models.

**Table 5:** Linear regression analysis of associations between waist-height ratio and depression/well-being scores–stratified by sex.

Depression/well-being scores	Model 1		Model 2		Model 3		Model 4	
**Males**	β (S.E.)	*P*	β (S.E.)	*P*	β (S.E.)	*P*	β (S.E.)	*P*
CES-D score	0.868 (.25)	**< .001**	0.965 (.25)	**< .001**	0.716 (.28)	**< .011**	0.593 (.29)	**.044**
WHO-5 score	-0.270 (.17)	.122	-0.424 (.17)	**.014**	-0.271 (.19)	.159	-0.163 (.20)	.417
**Females**	β (S.E.)	*P*	β (S.E.)	*P*	β (S.E.)	*P*	β (S.E.)	*P*
CES-D score	0.991 (.27)	**< .001**	1.115 (.27)	**< .001**	1.014 (.29)	**< .001**	0.768 (.30)	**.01**
WHO-5 score	-0.359 (.18)	**.041**	-0.513 (.17)	**.003**	-0.396 (.19)	**.034**	-0.292 (.19)	.128

Model 1: univariate.

Model 2: adjusted for age.

Model 3: adjusted for age, education, smoking status, alcohol use, physical activity and dietary quality.

Model 4: additionally adjusted for disease conditions (type 2 diabetes, cardiovascular disease and cancer).

Unstandardised β coefficients and standard errors (S.E) are shown. Significant *p* in **bold**.

As stratified analyses indicated stronger associations between adiposity measures and CES-D scores in females, we tested interaction terms in fully adjusted models using the full sample. No evidence of effect modification was observed (BMI*sex, *p* = .496; waist-height ratio*sex, *p* = .516).

## Discussion

In this cross-sectional study of 1,821 middle- to older-aged men and women in Ireland we demonstrate significant associations between measures of adiposity and psychological health, confirming our hypothesis that increased adiposity is related to mental health. Specifically, we examined BMI and waist-height ratio relationships with CES-D depression and WHO-5 well-being scores. These significant associations were retained following adjustment for demographic variables and lifestyle factors. Once disease conditions were accounted for, BMI and waist-height ratio relationships with the CES-D score remained and were more pronounced in females compared to males. Collectively these results suggest that increased adiposity is significantly associated with mental health, independent of lifestyle factors and disease conditions.

Our study findings are consistent with previous research [29–33]. A systematic review and meta-analysis of longitudinal studies reported that obesity increased the risk of onset depression [30]. A possible reason for this is that a high BMI often negatively affects self-esteem, self-image and body satisfaction–all known risk factors for depression [34]. An increase in weight can also exacerbate depression through social means, such as prejudice, discrimination and self- and societal-stigma [29,35]. The chronic pain that is directly caused by obesity, such as joint pain, back pain and fibromyalgia, is also known to result in depressive symptoms [36].

Obesity and depression are also thought to share similar biological pathways, namely through neurotransmitter imbalances, hypothalamus–pituitary–adrenal axis disturbances, dysregulated inflammatory pathways, increased oxidative and nitrosative stress, mitochondrial disturbances and neuroprogression, resulting in neurodegeneration [37]. Other biological factors that have been implicated in the obesity-depression link include genetics, alterations in other systems involved in homeostatic adjustments (neuroendocrine regulators of energy metabolism including leptin, insulin and the microbiome) and brain circuitries integrating homeostatic and mood regulatory responses [38].

Low socioeconomic status in childhood has been shown to be a strong predictor of depression in adulthood for a variety of reasons including material hardship, family disruption and an increased likelihood of stressful life events [39,40]. Importantly, these factors and poorer mental health may also lead to weight gain. The bidirectional association between depression and obesity was highlighted when Jantaratnotai et al. concluded that the treatment of one condition (depression or obesity) improves the prognosis of the second condition [41]. Consequently, as this is a cross-sectional study, it is possible that increased adiposity may be a consequence of poorer mental health and not a cause. Ha et al. constructed pseudopanel data to overcome the endogeneity problem when examining the effect of obesity on depression. It was found that obesity increased the likelihood of being depressed to a statistically significant extent. However, it was acknowledged that further research is required to overcome the reverse causality bias [42].

Although we did not observe evidence of effect modification in analyses, our study finding showing a more pronounced effect in females than in males is in agreement with some previous studies [43–47]. It has been shown that depression and anxiety diagnoses are approximately double for obese women compared to obese men [47–49]. Depressed females are more likely to have a raised appetite and subsequent weight gain and obesity has been found to be a better predictor of depression in females compared to their male counterparts [50,51]. In a study on sustained obesity and depressive symptoms, Carter and Assari found that the association between high BMI and depressive symptoms was positive and significant for White women, but non-significant for Black women [45]. It should be noted that the majority of our participants were Caucasian-Europeans due to the homogeneity of the Irish population. Also noteworthy in the same study was the finding that the association between high BMI and depressive symptoms was not significant for all males [45], contrasting to our own findings and other research studies on the relationship between obesity and depression in older males [52,53]. However, results from an Australian study which examined over 12,000 male participants between 65–84 years of age found that obesity was associated with an increased risk of incident depression among older men [54].

Research on the determinants of mental health is important for public health interventions and to gain a better insight into disease causation. Globally, the cost of mental health conditions has been estimated at more than US$16 trillion between 2010 and 2030 [55]. As our results imply that increased adiposity is significantly associated with mental health, independent of lifestyle factors and disease conditions, these findings suggest that targeted interventions for reducing depression should include better weight management population-level measures, particularly in middle- to older-aged populations.

This study has several strengths. The use of validated depression and well-being scales minimise outcome misclassification bias as our assessment of mental health did not rely solely on participants’ subjective reports or on their previous attendance to relevant health services. In addition, our measures of adiposity encompassed two indices to reduce exposure misclassification bias. Other strengths include equal representation by sex (49% male) and the availability of clinical data.

However, several limitations should be noted. As previously discussed, cross-sectional data precludes examination of temporal relationships leading to reverse causation bias and the lack of a causal inference. It is also possible that residual confounding is an issue in this study due to unmeasured and unknown additional potential confounders. Another limitation of this research is that our data were collected from a single primary care-based sample that may not be representative of the general Irish population. However, Ireland represents a generally ethnically homogenous population, so external validity may be less of an issue [56]. Additionally, previous research has estimated that 98% of Irish adults are registered with a GP and that even in the absence of a universal patient registration system, it is feasible to conduct population-based epidemiological studies that are representative using our methods [57].

## Conclusions

In conclusion, our study demonstrates a significant association between increased adiposity and poorer mental health in a middle- to older-aged population which is in agreement with previous evidence. In addition, these findings suggest that the positive relationship between adiposity and depression is independent of lifestyle factors and disease conditions. Targeted interventions for reducing depression should include better weight management population-level measures.

## Supporting information

S1 File Dataset. The Cork and Kerry Diabetes and Heart Disease Study dataset.
(XLSX)
